# U.S. adults who formerly owned firearms: 5-year incidence of and reasons for divestment among a national sample

**DOI:** 10.1186/s40621-026-00679-0

**Published:** 2026-04-09

**Authors:** Mary S. Lee, Deborah Azrael, Matthew Miller

**Affiliations:** 1 Department of Criminology, Law and Society, University of California, Irvine, Irvine, CA USA; 2 Harvard T.H. Chan School of Public Health, Boston, MA USA; 3 Bouve College of Health Sciences, Northeastern University, Boston, MA USA

**Keywords:** Firearm divestment, Firearm ownership dynamics, Firearm injury prevention

## Abstract

**Background:**

National surveys routinely provide estimates of personal and household firearm ownership, allowing changes in firearm prevalence to be assessed over time. By contrast, the determinants of changes in prevalence (new acquisitions and divestments) are rarely assessed. This study estimates the proportion of United States adults who divested of their firearms, describes divestor characteristics, and assesses why former gun owners chose to divest.

**Findings:**

Using data from the 2024 National Firearms Survey (*n* = 12,909), we estimate that 1.7% of United States adults (95% Confidence Intervals: 1.5, 2.0), voluntarily divested of their firearms in the 5-years prior to the survey. Of these 5.8 million divestors, an estimated 18.1% (95% Confidence Intervals: 12.5, 25.5) divested because of safety concerns. Overall, 23.6% (95% Confidence Intervals: 17.2, 31.6) of divestors currently live in a household with a gun owner.

**Conclusion:**

Nearly 6 million adults in the United States divested of their firearms in the 5-years prior to our survey; only one in five did so out of safety concerns. Future work should seek to understand more about the circumstances surrounding divestment from both those who viewed their firearms as posing a safety risk and from those who divested for other reasons.

## Introduction

National surveys routinely provide estimates of personal and household firearm ownership, allowing changes in firearm prevalence to be assessed over time. By contrast, the determinants of changes in prevalence (new acquisitions and divestments) are rarely assessed. An emerging literature focuses on new acquisition [1–5]. Far less is known about how often people divest of their firearms, the reasons they do so, or the characteristics of divestors. Information about divestment has the potential to provide additional context about the dynamics of firearm ownership, and to inform ongoing efforts to reduce firearm access to those at elevated risk of firearm injury.

The only national study of firearm divestment in the peer-reviewed literature is a survey conducted in 2015 that assessed divestment indirectly. This study estimated that 2% of adults in the United States (U.S.) who lived in a household with no firearms had, in the prior 5-years, lived in a household with firearms and subsequently chose to “sell or give away” their guns [6]. Of these divestors, 8% said that they had done so for “safety reasons.” More than half (55%) of divestors were women, and 65% were 65 years of age or older. The only other descriptive study of which we are aware to assess divestment used administrative data about handgun transfers in California; it found that 4.5% of *new* handgun owners divested within 5-years of their first acquisition [7]. Neither study distinguished voluntary from involuntary divestment, or assessed if the divestor lived in a household with firearms after divesting.

The current study extends earlier descriptive work using data from the 2024 National Firearm Survey (NFS). We use a direct measure of divestment, elicit information about whether divestment was voluntary or not, and, for voluntary divestors, explore the reasons they divested and whether they currently live in a household with another firearm owner.

## Methods

### Design and sampling

Data for this study come from the 2024 NFS, a national web-based survey designed by the authors (DA and MM) and conducted in December 2024 by the research firm Ipsos (described in detail elsewhere) [8]. Respondents were drawn from Ipsos’ KnowledgePanel (KP), an online panel that includes approximately 60,000 U.S. adults. The KP panel is selected on an ongoing basis using an equal probability of selection design to provide samples that are representative of the U.S. population. The study target population comprised non-institutionalized adults, age 18 and over, residing in the U.S.

A total of 21,907 KP members received an invitation to participate by email. One reminder email was sent to non-responders three days after initial outreach. Overall, 12,909 respondents completed the survey (survey completion rate = 59%). All of these respondents were asked to indicate whether they personally owned a gun and, separately, whether anyone else in their household owned a gun. Respondents who indicated that they did not personally own a gun were then asked the following sequence of questions to identify divestors: “You indicated that you don’t currently own a working gun. As an adult (e.g., since you were 18), was there any time in the past when you did personally own a gun?” followed by a question about how many years ago the respondent last owned a gun. Respondents who indicated that they had last owned a gun within the 5-years prior to the survey were asked why they had divested of their guns. Item response options were: “I no longer needed them;” “So that I or someone in my household no longer had access to the guns (e.g., an elderly parent, someone going through a hard time);” “For some other reason.” Those answering “for some other reason” were asked to describe their reason in free text.

### Analysis

Stata version 18.5 and its svy suite were used to generate descriptive statistics and 95% Confidence Intervals (CI). Survey weights, provided by Ipsos, adjusted for nonresponse and oversampling based on expected distributions from the U.S. Current Population and American Community Surveys.

## Results

4,059 respondents identified as a current firearm owner (28.6%; 95% CI: 27.8, 29.5). Of the 8,800 non-owners, 946 (an estimated 10.8% of U.S. adults, not shown) reported having ever owned a firearm. Of these, 225 reported having owned within the prior 5-years. Review of “For some other reason” free text found that 23 respondents had not divested voluntarily (e.g., they had been court ordered to divest or moved to a place where guns were not allowed, such as an assisted living facility). These 23 respondents were excluded from our analyses. Thus, a total of 202 reported voluntarily divesting within the past 5 years. These respondents represent an estimated 1.7% (95% CI: 1.5, 2.0) of U.S. adults, or 5.8 million adults.

Past 5-year voluntary divestors were more likely than current gun owners to be female (45.4%; 95% CI: 37.7, 53.4), Black (19.1%; 95% CI: 13.1, 26.9), younger (aged 18 to 44) (56.9%; 95% CI: 40.9, 71.7), and to be unmarried (60.2%; 95% CI: 46.5, 75.9) (Table 1). Among current gun owners, roughly half (47.0%; 95% CI: 45.2, 48.7) lived with another gun owner at the time of the survey, compared to a quarter of divestors (23.6%; 95% CI: 17.2, 31.6).

**Table 1:** Characteristics of current gun owners and of divestors who had owned firearms within the past 5-years: estimated distribution in numbers and weighted percentages with 95% CI

	Gun owners (*n* = 4059)^a^	Divestors (*n* = 202)^b^
Age		
18–29	231 (12.2%) [10.7, 13.8]	24 (22.8%) [15.8, 31.7]
30–44	837 (25.8%) [24.2, 27.4]	55 (32.1%) [25.1, 40.0]
45–59	1112 (26.3%) [24.9, 27.8]	32 (14.2%) [10.0, 19.8]
60+	1879 (35.7%) [34.2, 37.3]	91 (30.9%) [24.7, 37.9]
Gender		
Male	2733 (65.6%) [63.9, 67.2]	111 (54.6%) [46.6, 62.3]
Female	1326 (34.4%) [32.8, 36.1]	91 (45.4%) [37.7, 53.4]
Race/ethnicity		
White, Non-Hispanic	3162 (72.8%) [71.1, 74.5]	136 (54.5%) [46.3, 62.5]
Black, Non-Hispanic	351 (10.6%) [9.6, 11.8]	28 (19.1%) [13.1, 26.9]
Other, Non-Hispanic	249 (5.6%) [4.8, 6.6]	14 (8.2%) [4.5, 14.5]
Hispanic	297 (11.0%) [9.7, 12.3]	24 (18.2%) [12.2, 26.3]
Education level		
No high school diploma	135 (6.0%) [5.0, 7.1]	20 (14.4%) [9.3, 21.6]
High school graduate	891 (27.1%) [25.5, 28.8]	54 (32.3%) [25.0, 40.5]
Some college or associate	1305 (31.8%) [30.2, 33.4]	65 (30.8%) [24.0, 38.5]
Bachelor’s degree or higher	1728 (35.1%) [33.6, 36.7]	63 (22.6%) [17.3, 28.8]
Income		
≤ $49,999	751 (17.7%) [16.4, 19.0]	68 (34.7%) [27.5, 42.7]
≥ $50,000	3308 (82.3%) [81.0, 83.6]	134 (65.3%) [57.4, 72.5]
MSA status		
Non-Metro	803 (20.2%) [18.8, 21.6]	27 (14.1%) [9.1, 21.0]
Metro	3256 (79.8%) [78.4, 81.2]	175 (86.0%) [79.0, 90.9]
Marital status		
Now married	2726 (65.1%) [63.5, 66.8]	88 (39.8%) [32.5, 47.7]
Previously married	715 (14.9%) [13.8, 16.0]	69 (27.7%) [21.5, 34.8]
Never married	618 (20.0%) [18.5, 21.6]	45 (32.5%) [25.0, 41.1]
Employment status		
Not working	1557 (33.2%) [31.7, 34.8]	89 (38.1%) [30.9, 45.9]
Working	2502 (66.8%) [65.2, 68.3]	113 (61.9%) [54.1, 69.1]
Presence of children		
No	3128 (69.2%) [67.5, 70.9]	147 (61.2%) [52.8, 69.0]
Yes	931 (30.8%) [29.1, 32.5]	55 (38.8%) [31.0, 47.2]
Currently lives with a gun owner		
No	2315 (53.0%) [51.3, 54.8]	165 (76.4%) [68.4, 82.8]
Yes^c^	1744 (47.0%) [45.2, 48.7]	37 (23.6%) [17.2, 31.6]
Reason for divestment^d^		
Safety concerns	NA	31 (18.1%) [12.5, 25.5]
No longer needed the gun	NA	95 (45.2%) [37.4, 53.2]
For some other reason	NA	74 (36.6%) [29.2, 44.7]

^a^ The 4059 respondents who identified as current gun owners represent an estimated 28.6% of US adults (95% CI = 27.8, 29.5)

^b^ The 202 recent divestors represent an estimated 1.7% (95% CI = 1.5, 2.0) of US adults

^c^ Of divestors who cited safety reasons, 32.2% lived with a gun owner (95% CI: 16.2, 53.8), compared with 21.8% of those who divested for other reasons than safety (95% CI: 15.0, 30.4)

^d^ Two respondents refused to answer reason for their divestment

Approximately one in five divestors (18.1%; 95% CI: 12.5, 25.5*)* cited safety reasons for divesting. Among these divestors, 32.2% (95% CI: 16.2, 53.8) reported living with a gun owner at the time of the survey, as did 21.8% of respondents who divested for reasons other than safety concerns (95% CI: 15.0, 30.4). Almost half, 45.2% (95% CI: 37.4, 53.2), of divestors who reported that they gave up their guns for reasons other than safety did so because they “no longer needed” the gun. More than one-third of divestors, 36.6% (95% CI: 29.2, 44.7), cited “For some other reason”; free text responses described these reasons, which included giving firearms away (e.g., transferring them to family members), financial reasons, and other unspecified explanations that did not clearly fall under any of the provided categories.

## Discussion

Our overall estimate of divestment (1.7%) is in line with prior survey research using less direct assessment, which estimated that 2% of U.S. adults in 2015 had divested over the prior 5-years, and, as in the current study, found that the great majority of these divestors did so for reasons other than concern that their firearms posed a safety risk for members of their household. A novel finding from the current study is that almost one quarter of divestors reported currently living in a household with a firearm owner.

Our findings should be interpreted with several limitations in mind. First, despite screening over 12,000 people, only 202 respondents identified as having voluntarily divested, limiting our power to detect differences beyond crude comparisons. Second, free text responses describing miscellaneous reasons for divesting were difficult to interpret because many provided only limited information. Third, we do not know anything about the process by which our respondents decided that they no longer needed their firearms, or, for the minority who divested because of safety concerns, what risks they perceived their guns posed, or, for that matter, to whom (e.g., themselves, another member of their household). Fourth, although we find that approximately one-quarter of divestors currently live in a household with a gun owner, including those who divested for safety reasons, we do not know whether a similar proportion lived with a gun owner immediately after they divested. Despite these limitations, learning more from former gun owners about their experiences divesting has the potential to inform ongoing counseling efforts in clinical settings and help shape population-level campaigns that aim to reduce firearm injury and death by reducing access to firearms.

## Conclusion

Divestment is uncommon, yet firearm ownership is sufficiently prevalent that nearly 6 million U.S. adults had divested of their firearms in the 5-years prior to our survey. Future work should seek to understand more about the circumstances surrounding divestment, both from former gun owners who came to view their firearms as posing risk to members of their household (perhaps including themselves), and also from the great majority of former owners who divested after reaching the conclusion that they no longer needed their guns.

## Data Availability

Not applicable. Data were collected by Ipsos and researchers should contact the corresponding author with specific data requests.

## Abbreviations

ML: Mary Lee
DA: Deborah Azrael
MM: Matthew Miller
U.S.: United States
NFS: National Firearms Survey
KP: KnowledgePanel
CI: Confidence Intervals
